# Self-Awareness Deficits of Cognitive Impairment in Individuals With Schizophrenia. Really?

**DOI:** 10.3389/fpsyt.2020.00731

**Published:** 2020-07-30

**Authors:** Stéphane Raffard, Cindy Lebrun, Sophie Bayard, Alexandra Macgregor, Delphine Capdevielle

**Affiliations:** ^1^ Univ Paul Valéry Montpellier 3, Univ Montpellier, EPSYLON EA 4556, Montpellier, France; ^2^ Service Universitaire de Psychiatrie Adulte, Hôpital de la Colombière CHU Montpellier, Montpellier, France; ^3^ Inserm, U1061, Montpellier, France; ^4^ University of Montpellier, Montpellier, France

**Keywords:** schizophrenia, cognitive complaint, self-awareness, cognitive functioning, heterogeneity

## Abstract

**Objective:**

Misestimation of cognitive functioning has been largely described in individuals with schizophrenia. There is large evidence that correlations between subjectively assessed cognitive functioning and objectively determined cognitive functioning are weak in non clinical individuals and may be more closely related to other psychoaffective or clinical factors than to objective neuropsychological functioning. Surprisingly, no study to date has compared the associations between cognitive complaint and objective measures of cognitive functioning in individuals with schizophrenia and healthy controls. The main objective of this study was to 1) compare cognitive complaint between individuals with schizophrenia and non clinical controls, 2) explore the relationships between cognitive complaint and psychoaffective and clinical factors in the clinical group and 3) compare the relationships between subjective awareness of cognitive functioning and objective neuropsychological assessment in individuals with schizophrenia and non-clinical participants.

**Method:**

In this study 30 individuals with schizophrenia and 20 non-clinical matched controls were included. In addition to objective cognitive measures and subjective cognition assessed by the Subjective Scale To Investigate Cognition In Schizophrenia, measures of psychotic symptoms, depression, and anxiety were included.

**Results:**

Schizophrenia patients reported higher cognitive complaints in comparison with controls. In individuals with schizophrenia, cognitive complaint subscores were differently associated with depression, anxiety, and negative symptoms. When depression was controlled for, the same number of correlations between self-rated measures of cognition and objective measures of cognition were found in both groups, but accuracy of self-assessment of cognition was lower in the schizophrenia group.

When the schizophrenia group was divided into a high cognitive complaint group (SZ High CC) and a low cognitive complaint group (SZ Low CC), findings indicated that self-assessment of cognition in the SZ high CC was highly accurate (correlations with large effect sizes). By contrast the SZ low CC group severely misjudge their cognition.

**Conclusion:**

A significant proportion of patients with schizophrenia can accurately estimate their cognitive skills. Self-awareness of cognitive deficits in individuals with schizophrenia is an heterogenous phenomenon and misestimation of cognitive functioning might have been overestimated, partly due to secondary psychoaffective factors. Caution is warranted before jumping to the conclusion that all individuals with schizophrenia misjudge their cognitive functioning.

## Introduction 

Metacognition can be considered as an umbrella term describing a broad set of processes relating to the development of self-awareness (1) and ability to self-assess and self-reflect upon one’s emotional and cognitive experiences and abilities [See (2) for a review and discussion of the term applied to the field of clinical psychology]. Metacognitive deficits are considered by many authors as a core deficit of the schizophrenia spectrum disorders (3, 4). Metacognition impairments have been explored in individuals with schizophrenia in a large set of processes including reasoning and memory biases (5), beliefs (6), autobiographical memory (7), or insight into illness (8), to name but a few. This latter metacognitive ability, also named clinical insight (8), has been particularly studied in schizophrenia mainly because poor awareness into illness is highly prevalent in this population (9), leading to poor prognosis, and in association with self-stigma to higher risk of suicide (10) and depression (11). In this study we will focus on metacognitive awareness of cognitive functioning corresponding to the adequacy between the subjective report of cognitive functioning and objective cognitive performances.

In comparison with clinical insight, relatively few studies have examined these associations in individuals with schizophrenia despite evidence that cognitive impairments in the disease are present in most of cognitive domains including working and episodic memories, attention processes, abstraction, and executive functioning (12, 13). Several measures have been previously used to measure self-report of cognitive functioning in individuals with schizophrenia. Among them the Cognitive Failures Questionnaire [CFQ; (14)], the Measure of insight into cognition-self-rated [MIC-SR; (15)], or the subjective scale to investigate cognition in schizophrenia [SSTICS, (16)] which is the most commonly used tool to assess self-awareness of cognitive functioning in this mental disorder (17, 18).

To date, only one meta-analysis has been published on this subject (18). Several findings emerged from this meta-analysis and can be summarized as follows: 1) individuals with schizophrenia reported higher cognitive complaints than nonclinical controls; 2) modest associations between measures of objective and subjective cognition were found (the higher for problem-solving domain) 3) these relationships were higher when the study used the SSTICS to measure self-reported cognitive functioning in comparison with other scales, and 4) a weak association was found between depressive symptoms and cognitive complaint. Interestingly, several studies have been published after this meta-analysis, reporting as a whole similar results (19–22). However, and surprisingly, none of these studies about metacognitive awareness of cognitive functioning in schizophrenia examined and compared correlations between measures of objective and subjective cognition in a comparison group of non-clinical controls. For all that, previous studies in non-clinical individuals have constantly reported weak correlations between subjectively assessed cognitive functioning and objectively determined cognitive functioning (*r* generally ranging from 0.20 to 0.30) (23–25). In addition, it has been suggested that subjective cognitive complaint are more likely related to psychoaffective variables such as depression and anxiety rather than to psychotic symptoms or concurrent cognitive impairments in schizophrenia (21, 26). Note that this association was also previously reported in aging (23) or neurological disorders (27).

Consequently, the main objective of this study is to 1) compare cognitive complaint between individuals with schizophrenia and non clinical controls, 2) explore the relationships between cognitive complaint and psychoaffective and clinical factors in the clinical group, and 3) compare the relationships between subjective awareness of cognitive functioning and objective neuropsychological assessment in individuals with schizophrenia and non-clinical participants.

## Materials and Methods

### Method

#### Participants

Thirty outpatients with schizophrenia and 20 healthy controls completed this study. Inclusion criteria for patients were: (a) age between 18 and 65 years, (b) a DSM-5 diagnosis of schizophrenia, (c) adequate proficiency in French. Exclusion criteria for all participants were: (a) known neurological disease, (b) history of learning disability/developmental disorder, or (c) substance abuse in the past month (other than cannabis or tobacco). Controls were recruited from the general population and had the additional exclusion criterion that they had no personal lifetime history of any psychosis or affective disorders diagnosis. Controls with a family member with bipolar or schizophrenia disorders were also excluded. The comparison control group was selected to match the schizophrenia group on sex, age, and education-level variables. The demographic data are shown in **Table 1**.

**Table 1:** Demographics and clinical characteristics of schizophrenia patients and controls.

	Schizophrenia (N = 30)	Controls (N = 20)	Statistics	P value
***Demographic data***				
Sex (female)	11	7	χ2 = 0.90	0.57
Age (years)	33.73 ± 9.48 [21–56]	32.95 ± 13.34 [18–53]	t = 0.24	0.80
Education Level (years)	11.46 ± 3.39 [6–22]	12.3 ± 2.20 [9–16]	t = −0,96	0.33
***Clinical variables***				
Duration of illness	9.15 ± 9.46 [2–34]	–	–	–
PANSS Total score	72.40 ± 12.11 [38–88]	–	–	–
Positive symptoms	16.63 ± 5.48 [7–26]	–	–	–
Negative symptoms	21.23 ± 5.89 [12–35]	–	–	–
General psychopathology	34.16 ± 6.45 [18–47]	–	–	–
BDI	16.30 ± 9.18 [0–32]	8.20 ± 4.16 [2–17]	t = 2.72	**0.009**
STAI-S	37.06 ± 11.36 [20–69]	33.30 ± 7.82 [23–47]	t = 1.06	0.29
STAI-T	41.10 ± 11.64 [20–72]	40.45 ± 9.44 [24–59]	t = 0.20	0.83
SSTICS				
Total score	25.56 ± 9.10 [1–46]	22.55 ± 7.87 [7–40]	F = 5.12	**0.02**
Working memory	3.03 ± 1.86 [0–7]	3.35 ± 1.87 [1–8]	F = 0.62	0.54
Explicit memory subscale	11.70 ± 3.38 [0–21]	9.35 ± 3.93 [3–15]	F = 6.14	**0.04**
Attention subscale	6.46 ± 4.23 [0–17]	6.20 ± 2.83 [1–12]	F = 2.05	0.13
Executive functions subscale	2.83 ± 2.32 [0–9]	2.20 ± 1.90 [0–7]	F = 1.63	0.20

N, sample size; Mean ± standard deviation [range]; BDI-II, Beck Depression Inventory; STAI-T, trait State-Trait Anxiety Inventory; STAI-S, state State-Trait Anxiety Inventory; PANSS, Positive and Negative Syndromes Scale total score; Bold font indicates a significant correlation (p < 0.05).

### Measures

#### Clinical Variables

In the schizophrenia sample, severity of symptoms was evaluated with the positive and negative syndrome scale (PANSS) for schizophrenia (28). For all participants current emotional status was assessed using the Beck Depression Inventory-II (BDI-II) (29) and the Spielberger State Trait Anxiety Inventory (STAI Trait and STAI State) (30).

#### Cognitive Complaint

##### Subjective Scale to Investigate Cognition in Schizophrenia (SSTICS)

Cognitive complaint was measured by the SSTICS (16). Twenty-one items focus on four cognitive domains: memory, attention, executive functions, and praxia. Each item is rated on a five-point Likert scale ranging from “never” to “very often” and refers to how often a problem occurs. A higher score suggests higher cognitive complaint. The scale has good internal consistency (α = 0.86) and test–retest reliability (r = 0.80; 16). For this study, we focused on working memory, explicit memory, attention, and executive functions subscales.

#### Objective Cognitive Measures

We used a computer-based neuropsychological battery, Zimmermann and Fimm (31)’s attention test battery (Testbatterie zur Aufmerksamkeitsprüfung—TAP) to evaluate executive and attentional processes. This well-validated test battery provides normative data for adults. The task consists of simple, easily distinguishable stimuli to which the patient’s motor response is recorded. A button box with millisecond accuracy was used to capture reaction times (RTs) and record responses (false alarms and/or omissions). Several subscales of the TAP were used and administered in random order: the 2-Back Task (*Updating*), the Go/No-Go Task (*Inhibition*), the Flexibility Task (*Shifting*), the Divided Attention Task (*Dual task coordination*), and the Vigilance Task (*Vigilance*) to assess executive and attentional processes. To assess verbal learning we used the Grober and Buschke verbal learning test (32).

#### Executive and Attentional Processes

##### Go/No-Go Task

This subtest measures the ability to suppress a response in the presence of irrelevant stimuli as well as the response latency during stimulus selection. In this study, a version of the Go-nogo task with a higher memory load with five stimuli, squares with different textures, was realized. One 3 × 3 cm square appears in the middle of the screen. There are two target and three nontarget stimuli. The subject has to press the button on the presentation of a target and not to press on the presentation of a nontarget. A total of 60 trials were presented. The median RT served as primary measure.

##### Flexibility Task

In this subtest the flexibility of focused attention is tested by a mental alternation between two sets of targets. There are two alternatives for testing, a verbal and a non-verbal version. In this study, we used the verbal version in which the sets of target consist of letters and numbers. For testing, two stimuli, one from each set, are presented simultaneously and randomly on the left or the right side of the fixation point. From one presentation to the other, the target changes from letter to number and *vice versa*. The subject has to press as quickly as possible the key on the side of the target (left or right). The dependent measure was the median RT. The dependent measure was the median RT.

##### The 2-Back Task

The 2-Back is a verbal working memory task in which series of consonants are presented visually, one every 3,000 ms (2,500 ms stimulus and 500 ms interstimulus interval). Participants were asked to make a yes/no response following each consonant, determining whether it was the same as or different from the consonant presented two earlier (e.g., f, N, b, N, B, K, b, k, N, G…). Responses were made using two keys on the keyboard of a laptop computer. Capitalization was randomized and each consonant block contained 33% targets. The effects of the 2-Back condition were obtained by comparing performance to the 0-Back, a control condition. The 0-Back task included nine consonants presented at the same rate, with 33% targets. Participants responded yes when a predetermined target consonant (“X” or “x”) appeared and no for other consonants. In total, there were four 6-min series of four 0-Back/2-Back cycles each. As the dependent variable, we computed the difference in RT between the 0-back and 2-back condition, which should reflect the specific working memory-related task demands (storage and updating).

##### Divided Attention Task

The divided attention task required the simultaneous performance of spatial and auditory tasks. The spatial task comprised the identification of a pre-determined visual configuration assembled from crosses. For the auditory task, the participants were instructed to detect a ‘‘beep/bop’’ alternation in a repetitive sequence. Number of omission errors and commission errors were recorded for further analysis.

##### Vigilance Task

This task assesses the subject’s ability to sustain attention for long periods of time. Vigilance was tested over a period of 30 min at a rate of one critical stimulus per minute This subtest presents the subject with a series of high and low tones (440 and 1,000 Hz) in regular alternation (“di da di da…”). The subject must discover the appearance of an irregularity in the sequence. The target stimuli have a low frequency of appearance. The median RT was the dependent variable.

##### Alertness Task

This task concerns simple RT measurements, in which a cross appears on the monitor at randomly varying intervals and to which the subject should respond as quickly as possible by pressing a key. The task also concerns the RT following the presentation of an audible warning signal, which allows the calculation of a phasic alertness index (improvement of the reaction speed following the presentation of the warning signal). The phasic alertness index was the dependent variable.

##### The Grober and Buschke Verbal Learning Test

The test is composed of 16 words belonging to 16 different semantic categories and presented on cards containing four words at a time (32). The patient is asked to read aloud and to point to each item (e.g. herring) following the examiner’s indication of a category (e.g., “what is the name of the fish?”). When the four items are thus learned, the card is removed and the patient is given the four categories as a cued immediate recall. If the patient is enable to respond, the card is shown again and the same steps as above are carried out until every item is encoded. The learning phase is achieved when all or nearly all the items have been encoded. The test includes therefore successively an encoding control phase, an immediate cued recall, then three successive free and cued recall trials, a delayed free and cued recall phase (20 min), followed by a recognition phase. For both free recall 3 and delayed free recall, the amount of correctly recalled items was registered.

### Procedure

Participants’ recruitment took place within the University Department of Adult Psychiatry in Montpellier CHU. All participants were tested individually in two sessions: the first session included a clinical assessment (PANSS, SSTICS, BDI-2, STAI) conducted by a fully trained clinical psychologist; 1 or 2 days following the first session, the second session was completed involving the administration of the neuropsychological tests by a fully trained clinical neuropsychologist. The clinical neuropsychologist was blind of the SSTICS scores of the participants. The study was approved by the relevant ethical review board [Comité de Protection des Personnes (CPP) Ile de France IX] on January 18, 2010. Written informed consent was obtained from all participants.

### Statistical Analyses

All statistical analyses were performed with IBM SPSS software version 24.0. The continuous variables were expressed as mean ± standard deviation and categorical variables as frequency and percentage. Prior to analysis, all data were examined for normality using the Shapiro–Wilk test. The Shapiro–Wilk tests revealed non-normal distributions for executive and attentional processes (p < 0.05). Therefore, the Kruskal–Wallis test was chosen as a non-parametric alternative to the analysis of variance tests. The median RT for all executive and attentional processes, the number of omission errors and commission errors for divided attention and the phasic alertness index were examined using *H* tests. *Post hoc* analyses were performed using the Mann-Whitney U test to determine the directions of the significant *H* test results. The significance level was set at p < 0.05. Furthermore, effect sizes (Cohen’s r) of all significant group differences were computed. Cohen’s r was chosen as it does not rely on the normality assumption. Based on Cohen’s criteria for r = 0.10 indicates a small effect, 0.30 indicates a medium effect, and 0.50 indicates a large effect (33).

All other variables exhibited normal distributions (p > 0.05). Therefore, we conducted parametric analyses of variance (ANOVAs) and Student’s t-tests to assess group differences for continuous variables. The Chi-square test was performed for categorical variables. In addition, we calculated the eta squared *η* ^2^ and the Cohen’s *d’* as measure of the effect size. The effect size was considered small (*η* ^2^ = 0.01; *d* = 0.2), medium (*η* ^2^ = 0.06; *d* = 0.5), or large (*η* ^2^ = 0.14; *d* = 0.8) according to Cohen (33).

To test for potential relationships between variables in groups, we calculated partial correlations (with rs of.10,.30, and.50 defined as small, medium, and large effect size, respectively, 33). In addition, bivariate correlations between cognitive complaint and clinical characteristics for schizophrenia patients were calculated according to Pearson’s p. The α-level was set to p = 0.05.

Finally, additional exploratory statistical analyses were performed in the clinical group of individuals with schizophrenia in order to compare objective measures of cognitive functioning regarding their level of cognitive complaint. For this, the schizophrenia group was divided into a high cognitive complaint group and a low cognitive complaint group, based on a median split of the SSTICS total score. We also explored the relationships between objective and subjective measures of cognitive functioning in these both subgroups of patients.

## Results

### Demographics

Groups did not differ significantly in terms of age (t _(48)_ = 0.24, p = 0.80), sex (χ2 = 0.90, p = 0.57), education level (t_(48)_= −0,96, p = 0.33), or state and trait anxiety (respectively, t_(48)_= 1.06, p = 0.29, t(48)= 0.20, p = 0.83). Level of depressive symptoms was significantly higher in schizophrenia patients than in control participants (t_(48)_= 2.72, p = 0.009, *d’* = 0.86). **Table 1** presents demographic and clinical characteristics of the participants.

#### Do Patients With Schizophrenia Differ From Control Participants on Cognitive Complaint?

##### Schizophrenia Patients Versus Control Group Comparisons

Given that patients and controls significantly differ on depressive symptoms, the BDI-II was considered as a covariate in the group comparison analysis. A multivariate ANCOVA was used to examine group differences in cognitive complaint (**Table 1**). The results showed significant difference on the SSTICS total (F = 3.99, p = 0.02, *η2* = 0.14) and explicit memory scores (F = 3.14, p = 0.04, *η2* = 0.11) between schizophrenia patients and control participants, indicating that patients have a higher cognitive complaint. No significant difference was noticed for the other cognitive domains (working memory, attention, and executive functions) rated on the SSTICS (all ps > 0.05).

#### Relationships of SSTICS Scores to Clinical Measures for Schizophrenia Participants

**Table 2** shows correlations between each domains of cognitive complaint and other clinical measures in patients.

**Table 2:** Pearson correlations between cognitive complaint domain and clinical measures for schizophrenia patients.

Clinical variables	SSTICS-total	SSTICS-working memory	SSTICS-explicit memory	SSTICS-attention	SSTICS-executive functions
Disease duration	0.56**	0.22	0.45**	0.48**	0.23
BDI-II	0.40*	0.13	0.48**	0.36**	0.26
STAI-S	0.27	0.42**	0.05	0.48**	0.16
STAI-T	0.62**	0.49**	0.33*	0.65**	0.46**
PANSS Total score	−0.23	−0.30	−0.06	−0.25	−0.31
Positive symptoms	−0.06	−0.08	−0.08	−0.06	−0.33
Negative symptoms	−0.40*	−0.33	−0.27	−0.37*	−0.30
General psychopathology	−0.08	−0.28	−0.09	−0.21	−0.06

SSTICS, Subjective Scale to Investigate Cognition in Schizophrenia; PANSS, Positive and Negative Syndromes Scale total score; BDI-II, Beck Depression Inventory, STAI-T, trait State-Trait Anxiety Inventory; STAI-S, state State-Trait Anxiety Inventory; *p <.05; **p <.01; (medium and large effect are shown in bold print)

##### Overall Cognitive Complaint

Higher overall cognitive complaint in schizophrenia patients is associated with longer disease duration, and higher levels of both depressive symptoms and trait anxiety. Higher overall cognitive complaint in schizophrenia patients is associated with lower levels of negative symptoms. The size of this association was moderate to large.

##### Complaint of Memory Problems

Higher complaint of working memory problems in schizophrenia patients is associated with higher levels of both state and trait anxiety. Higher complaint of explicit memory problems in schizophrenia patients is associated with longer disease duration and higher levels of both depressive symptoms and trait anxiety. The size of this association was moderate.

##### Complaint of Attention Problems

Higher complaint of attention problems in schizophrenia patients is associated with longer disease duration and higher levels of depressive symptoms and both state and trait anxiety. Higher complaint of attention problems in schizophrenia patients is associated with lower levels of negative symptoms. The size of this association was moderate to large.

##### Complaint of Executive Problems

Higher complaint of executive problems in schizophrenia patients is only associated with higher levels of trait anxiety. The size of this association was moderate.

#### Relationships of SSTICS Scores to Objective Cognitive Measures for Schizophrenia Patients and Controls

As mentioned above, groups differ according severity of depressive symptoms rated on the BDI. The following correlational analyses were therefore controlled for depressive symptoms.

##### Schizophrenia Patients

Higher overall cognitive complaint in s*chizophrenia patients* was significantly associated with lower phasic attention (r = .39, p = 0.034)

Higher complaint of explicit memory problems in s*chizophrenia patients* was significantly associated with lower phasic attention (r = 0.46, p = 0.013) and lower vigilance performances (r = −.34, p = 0.029).

Higher complaint of working memory problems in s*chizophrenia patients* was significantly associated with lower phasic attention (r = .34, p = 0.032) and higher updating deficits (r = .45, p = 0.017). Overall, the size of these associations was moderate.

##### Controls

Higher overall cognitive complaint *in controls* was significantly associated with lower inhibition performances (r = −0.49, p = 0.014).

Higher complaint of explicit memory problems in *controls* was significantly associated with lower memory performances (r = −0.43, p = 0.030).

Higher complaint of working memory problems in *controls* was significantly associated with lower higher updating deficits (r = .44, p = 0.026)

Higher complaint of executive problems *in controls* was significantly associated with lower inhibition performances (r = −0.46, p = 0.020).

Higher complaint of attention problems in *controls* was significantly associated with lower inhibition performances (r = −0.41, p = 0.034). Overall, the size of these associations was moderate.

#### Additional Exploratory Analyses

##### Schizophrenia Patients With High and Low Cognitive Complaint versus Control Group Comparisons

The schizophrenia group was divided into a high cognitive complaint (SZ High CC) group and a low cognitive complaint group (SZ Low CC), based on a median split of the SSTICS total score. Further analysis of cognitive complaint domains was thus performed on 14 SZ High CC, 16 SZ Low CC, and 20 controls. These groups did not differ in terms of age (F = 2,46, p = 0.09), sex (χ2 = 2.04, p = 0.36), education level (F = 0.47, p = 0.62), or both state (F = 1.05, p = 0.35) and trait anxiety scores (F = 2,27 p = 0.11). However, the groups differed on BDI-II score (F = 4.15 p = 0.02). SZ High CC (M = 19.78 ± 7.66) had significantly higher depressive symptoms compared to both controls (M = 9.20 ± 5.13, p <.001) and SZ Low CC groups (M = 13.25 ± 9.53, p = 0.04). However, SZ Low CC and controls did not differ. The comparison analyses were therefore controlled for depressive symptoms.

**Table 3** displays clinical data for SZ High CC and SZ Low CC groups. SZ High CC and SZ Low CC groups differed in terms of age (t = −2,77, p= 0.01), disease’s duration (t = −4,39, p < 0.001), and severity of depressive symptoms (t = −2.04, p = 0.04).

**Table 3:** Demographics and clinical characteristics of schizophrenia patients regarding their level of cognitive complaint.

	High cognitive complaint * (N = 14)	Low cognitive complaint *(N = 16)	Statistics	P value
***Demographic data***				
Sex (female)	7	4	χ2 = 2.04	0.36
Age (years)	36.35 ± 10.30 [27–56]	29.68 ± 6.67 [21–46]	t = −2,77	**0.01**
Education Level (years)	11.35 ± 3.02 [6–17]	11.56 ± 3.77 [7–22]	t = 0.16	0.87
***Clinical variables***				
Duration of illness	15.05 ± 10.37 [3–34]	3.59 ± 3.03 [2–9]	t = −4,39	**<0.001**
BDI	19.78 ± 7.66 [0–37]	13.25 ± 9.53 [0–26]	t = −2.04	**0.04**
STAI-S	38.42 ± 11.95 [20–69]	35.87 ± 11.07 [20–54]	t = −0.60	0.54
STAI-T	45.42 ± 10.52 [20–72]	37.31 ± 11.54 [20–52]	t = −2.00	0.05
PANSS Total score	71.28 ± 10.31 [54–87]	73.37 ± 13.75 [38–88]	t = −0.46	0.64
Positive symptoms	16.55 ± 5.37 [9–26]	16.68 ± 5.74 [7–26]	t = −0.05	0.95
Negative symptoms	20.14 ± 4.68 [13–28]	22.18 ± 6.79 [12–35]	t = −94	0.35
General psychopathology	33.78 ± 6.60 [20–47]	34.50 ± 6.53 [18–44]	t = −0.29	0.76

N, sample size; Mean ± standard deviation [range]; BDI, Beck Depression Inventory; STAI-T, trait State-Trait Anxiety Inventory; STAI-S, state State-Trait Anxiety Inventory; PANSS, Positive and Negative Syndromes Scale total score; Bold font indicates a significant correlation (p<0.05)

*The schizophrenia group was divided into a high cognitive complaint (SZ High CC) group and a low cognitive complaint group (SZ Low CC), based on a median split of the SSTICS total score.

#### Do Schizophrenia Patients High and Low Cognitive Complaint Groups Differ Across Objective Cognitive Measures?

Kruskal–Wallis tests revealed significant differences between patients with SZ and control participants on objective cognitive measures. The *Post hoc* comparisons (Mann-Whitney U tests) of the SZ groups to controls and corresponding effect sizes indicated impairments on individual test measures ranging from medium to large effect sizes. In comparison to the control participants, the SZ Low CC and SZ High CC groups showed significantly poorer performances with regard to inhibition processes (large effects for Go/no-go RT), flexibility, (large effects for flexibility RT), storage and updating (medium to large effects for n-back RT), as well as for attentional processes (medium effects for vigilance RT and large effects for the divided attention omissions errors), and memory (large effects for free recall 3 and delayed free recall). The groups did not differ on phasic alertness. Considering subgroups of patients’ analyses, the SZ Low CC and SZ High CC groups did not differ significantly from each other on any of the neuropsychological measures (**Table 4**).

**Table 4:** Objective cognitive measures performances by group regarding the level of cognitive complaint schizophrenia patients and control participants.

Variables	High cognitive complaint*(N = 14)	Low cognitive complaint*(N = 16)	Controls(N = 20)	Kruskal–Wallis *H* test	***Post hoc*** U test		
	Mean ± standard deviation	Mean ± standard deviation	Mean ± standard deviation	P Value	High cognitive complaint *vs* Low cognitive complaint(Cohen’s r)	Controls *vs* High cognitive complaint(Cohen’s r)	Controls *vs* Low cognitive complaint(Cohen’s r)
Go/no-go task (RT)	673.42 ± 106.99	656.50 ± 120.50	553.52 ± 61.74	**p = 0.001**	U = 96 n.s.	U = 43, **p = 0.001** (0,58)	U = 67, **p = 0.003** (0,50)
Flexibility task (RT)	1315.75 ± 430.76	1234.75 ± 353.12	700.17 ± 124.69	**p < 0.001**	U = 102 n.s.	U = 22, **p < 0.001** (0,70)	U = 31, **p < 0.001** (0,70)
The 2-back Task (RT)	312.32 ± 465.44	230.28 ± 232.60	93.50 ± 104.93	**p = 0.024**	U = 108 n.s.	U = 86, **p = 0.049** (0,55)	U = 99, **p = 0.047** (0,33)
Divided attention task							
Omissions	4.85 ± 4.31	5.25 ± 6.38	0.95 ± 1.05	**p = 0.001**	U = 108 n.s.	U = 46.5, **p < 0.001** (0,57)	U = 67, **p = 0.002** (0,52)
Commissions	3.14 ± 5.34	2.25 ± 2.95	0.85 ± 1.08	n.s.	–	–	–
Vigilance task(RT)	627.31 ± 194.57	624.56 ± 116.64	525.22 ± 77.35	**p = 0.010**	U = 106 n.s.	U = 69, **p = 0.013** (0,42)	U = 84, **p = 0.016** (0,41)
Alertness task (Phasic alertness index)	0.0592 ± 0.21	0.0005 ± 0.13	0.0069 ± 0.06	n.s.	–	–	–
The Grober and Buschke verbal learning test							
Free recall 3	11.35 ± 2.30	11.18 ± 2.22	14.25 ± 1.74	**p < 0.001**	U = 110 n.s.	U = 45.5, **p = 0.001** (0,57)	U = 39.5, **p < 0.001** (0,66)
Delayed free recall	10.35 ± 3.05	10.56 ± 2.52	14.60 ± 1.35	**p < 0.001**	U = 107, 5 n.s.	U = 24.5, **p < 0.001** (0,71)	U = 20, **p < 0.001** (0,77)

N = sample size; Post hoc U tests were computed only for significant effects on the H test; Bold font indicates a significant correlation (p < 0.05)

*The schizophrenia group was divided into a high cognitive complaint (SZ High CC) group and a low cognitive complaint group (SZ Low CC), based on a median split of the SSTICS total score.

#### Relationships of SSTICS Scores to Objective Cognitive Measures for Schizophrenia Patients High and Low Cognitive Complaint Groups

As described above in **Table 3**, SZ High CC and SZ Low CC groups differed in terms of age, disease’s duration, and severity of depressive symptoms. The following correlational analyses were therefore controlled for age, disease’s duration, and depressive symptoms.

#### Schizophrenia High Cognitive Complaint

Higher overall cognitive complaint in SZ High SA was significantly associated with lower updating deficits (r = .66, p = 0.028).

Higher complaint of explicit memory problems in SZ High SA was significantly associated with lower memory abilities (r = −0.67, p = 0.024).

Higher complaint of working memory problems in SZ High SA was significantly associated with higher inhibition deficits (r = −0.63, p = 0.037). Overall, these relationships have large effect sizes.

##### Schizophrenia Low Cognitive Complaint

There were no significant correlations between SSTICS scores and objective cognitive measures.

## Discussion

The aim of our study was to 1) compare cognitive complaint between individuals with schizophrenia and non clinical controls, 2) explore the relationships between cognitive complaint and psychoaffective and clinical factors in the clinical group, and 3) compare the relationships between subjective awareness of cognitive functioning and objective neuropsychological assessment in individuals with schizophrenia and non-clinical participants

First, our findings indicated that at group level individuals with schizophrenia reported higher subjective cognitive difficulties when depression was controlled for regarding some (SSTICS total score and the explicit memory subscore) but not all SSTICS subscores. Indeed, no differences between groups were found for working memory, attention, and executive functions subscores of the SSTICS. Concerning increased subjective cognitive complaint in patients in comparison with controls, this result is in accordance with the findings of the meta-analysis of Potvin et al. (18) who have observed a similar results for the SSTICS total score.

Regarding the second aim of our study, significant relationships were found between psychoaffective measures (state anxiety and depression), duration of illness, psychotic symptoms (i.e. negative symptoms), and cognitive complaint in the clinical group. Regarding psychoaffective factors this is in accordance with some (20, 21, 26, 34) but not all studies on this subject (35, 36) in schizophrenia. In our study, and similarly to the study of Bayard et al. (26) we used the Beck depression Inventory 2 which is a self-rated measure of depression whereas Potvin et al. (36) for example used the Calgary Depression Scale for Schizophrenia which consist on a semi-structured interview (37). Similarly, the STAI is also a self-reported measure of anxiety. Taken together, this can explain our findings and confirm the view that subjective cognitive complaints are related to depression and psychoaffective variables in individuals with schizophrenia as it has been consistently found in other populations such as chronic pain (38), older adults (39 for a review), neurological disorders such as multiple sclerosis (40) or mild cognitive impairment (41) particularly when self-reported measures of depression or anxiety are used.

For the third aim of our study and as the two groups (controls *vs.* individuals with schizophrenia) differed according to the severity of depressive symptoms, we computed partial correlations between the subtests of the SSTICS and the corresponding objective neuropsychological measures controlling for depression. For the overall sample of schizophrenia patients, five significant correlations with a moderate effect size were found. Similarly regarding the control group, five significant correlations were found, also with a moderate effect size. However, whereas significant correlations were related to three cognitive domain in patients (overall cognitive functioning, working memory, and explicit memory), five scores of the SSTICS were associated with objective cognitive measures in controls suggesting better and more accurate self-awareness of cognitive functioning in healthy controls in comparison with individuals with a diagnosis of schizophrenia.

However, we decided to further explore the relationships between subjective measure of cognition and objective cognition in the schizophrenia group. The schizophrenia group was divided into a high cognitive complaint (SZ High CC) group and a low cognitive complaint group (SZ Low CC), based on a median split of the SSTICS total score. If the two groups did not differ significantly from each other on any of the neuropsychological measures, they differed in terms of age, disease’s duration, and severity of depressive symptoms, the SZ High CC group having higher depressive symptoms and a longer duration of illness than the SZ low CC. We found in the SZ High CC group three correlations between the SSTICS scores and objective cognitive functioning with large effect sizes. It is important to note that even if five correlations were found in the control group, effect sizes were only moderate. Additionally, as in the control group the SSTICS scores matched adequately with the objective neuropsychological measures (i.e. explicit memory subscore of the SSTICS with the Z-score of the Grober and Buscke verbal learning test and inhibition with working memory subscore of the SSTICS). It is important to note that unlike to past previous studies, neuropsychological tools were chosen in our study to fit adequately and specifically with the different SSTICS subsets in order that each test can correspond with a domain evaluated by the SSTICS. In our study we used a relatively large number of specific cognitive processes compared to other past studies on this topic which often used measures of global functioning. It is possible that the convergent validity of our cognitive assessment with the SSTICS might have increased the validity of participant’s estimation of their own cognitive functioning with actual performance ranking.

Finally, no correlations between SSTICS scores and any of the neuropsychological scores were found in the low SZ CC group. This suggests that if some patients exhibit a good self-awareness of cognitive functioning, a significant proportion have clear neurocognitive insight deficits. Nevertheless and from a clinical perspective, several studies indicated that impaired neurocognitive insight is not a barrier to participate to a cognitive remediation program. For example Burton & Twamley (19) showed that among individuals with impaired neurocognitive insight, those who participated to a cognitive remediation program performed better than those who did not receive treatment. Furthermore, rather than focusing on cognitive complaint (strongly associated with depression and emotional distress), focusing on functional disabilities could be a better pathway to promote a personalized approach leading to sustainable functional changes (42), particularly in individuals with impaired abilities to recognize and manage one’s own cognitive functioning.

Taken together, our results suggest that even if we must acknowledge that the schizophrenia group as a whole exhibits relatively poorer self-awareness of cognitive functioning in comparison to the non-clinical group, one cannot conclude that they have strong self-awareness deficits. Indeed, a similar number of correlations between subjective scores of the SSTICS and objectives measures of cognition with moderate to large effect sizes were found in the two groups. In this context, the state that individuals with schizophrenia « did a very poor job of accurately classifying their cognitive status » compared to healthy controls (43) is inaccurate and an oversimplification of the neurocognitive insight phenomenon. Indeed, our findings clearly indicate that schizophrenia patients should not be considered as an homogeneous group as it is too often the case. When we split our clinical group in a high and a low cognitive complaint groups, individuals in the SZ High CC group had accurate and high self-awareness of their cognitive status, and even larger to what it is usually reported in healthy controls (44). As a consequence, if there is good evidence for significant cognitive heterogeneity in schizophrenia (45), it appears that subjective cognition in schizophrenia is also clearly heterogeneous and the extent of heterogeneity is yet to be investigated in larger samples.

From a theoretical perspective, and even if metacognition deficits commonly occur in schizophrenia (2), our findings support the view that some individuals with schizophrenia have preserved metacognitive abilities. As pointed out by some authors (3, 4), an individual’s capacity for metacognition cannot be assess categorically or dichotomize as present or absent. Instead, similarly to nonclinical individuals, metacognition is a multidimensional construct varying in individuals with schizophrenia along several and independent measurable dimensions.

Our study has some limitations. One of the main limitations in the present study is the small sample size of our two groups of participants. In addition, we decided to divide the schizophrenia group (n = 30) into those with high and low cognitive complaints. Even if we controlled for potential confounds, we cannot exclude that we have produced false-positive results, and/or over-estimated the magnitude of the associations found in our study. Thirdly, the use of a median split approach for subdividing patients into subgroups of high and low cognitive complaint is not optimal. Future studies should perform correlational analyses between objective and subjective cognitive functioning in subgroups of individuals with schizophrenia patients regarding their cognitive complaint in a larger sample and based on well-defined cut-off values on the SSTICS. Fourth, anxiety was assessed with the STAI in our two groups, which is not optimal to measure anxiety, even more so in individuals with schizophrenia. Further studies involving well-validated scales such as the Scale of Anxiety Evaluation are needed (46). Finally, there is now evidence that outside psychoaffective states performance on measures of neurocognition in schizophrenia are to a considerable extent due to secondary factors such as poor motivation, fatigue, and other momentary impairments (47). These variables should be taken into account in future studies.

## Conclusion

In summary, our results suggest that a significant proportion of patients with schizophrenia can accurately estimate their cognitive skills. Misestimation of cognitive functioning might have been overestimated in individuals with schizophrenia, partly due to psychoaffective confounding factors. The findings of this study confirm the heterogeneous and complex nature of self-awareness and metacognitive abilities in this mental disorder. Caution is warranted before jumping to the conclusion that all individuals with schizophrenia misjudge their cognitive functioning.

## Data Availability Statement

The datasets generated for this study are available on request to the corresponding author.

## Ethics Statement

The studies involving human participants were reviewed and approved by Comité de Protection des Personnes (CPP) Ile de France IX on January 18, 2010. The patients/participants provided their written informed consent to participate in this study.

## Author Contributions

SR conceived and designed the experiments. SR and DC conducted the experiments and collected data. CL, SB analyzed the results. SR and CL wrote the main manuscript text. All authors contributed to the article and approved the submitted version.

## Conflict of Interest

The authors declare that the research was conducted in the absence of any commercial or financial relationships that could be construed as a potential conflict of interest.
